# Readability of web-based sources about induced abortion: a cross-sectional study

**DOI:** 10.1186/s12911-020-01132-y

**Published:** 2020-06-05

**Authors:** Susanne Georgsson, Tommy Carlsson

**Affiliations:** 1 The Swedish Red Cross University College, Huddinge, Sweden; 2 Department of Clinical science, Intervention and technology, Karolinska Institutet, Stockholm, Sweden; 3 Department of Women’s and Children’s Health, Uppsala university, MTC-huset, Dag Hammarskjölds väg 14B, 1 tr, SE-75237 Uppsala, Sweden

**Keywords:** Consumer health information, Induced abortion, Readability, World wide web, Quality

## Abstract

**Background:**

High-quality information is essential if clients who request an abortion are to reach informed decisions and feel prepared for the procedure, but little is known concerning the readability of web-based sources containing such material. The aim was to investigate the readability of web-based information about induced abortion.

**Methods:**

The search engine Google was used to identify web pages about induced abortion, written in the English language. A total of 240 hits were screened and 236 web pages fulfilled the inclusion criteria. After correcting for duplicate hits, 185 web pages were included. The readability of the text-based content of each web page was determined with Flesch Kincaid Grade Level, Gunning Fog Index, Coleman-Liau Index, Simple Measure of Gobbledygook, and Flesch Reading Ease. Data were analyzed with descriptive statistics, Pearson’s correlation coefficient and Kruskal-Wallis with Dunn’s test as post hoc analysis.

**Results:**

Across all grade level measures, a small minority of the web pages had a readability corresponding to elementary school (*n* < 3, 1%), while the majority had readability corresponding to senior high school or above (*n* > 153, 65%). The means of the grade level measures ranged between 10.5 and 13.1, and the mean Flesch Reading Ease score was 45.3 (SD 13.6). Only weak correlations (rho < 0.2) were found between the readability measures and search rank in the hit lists. Consistently, web pages affiliated with health care had the least difficult readability and those affiliated with scientific sources had the most difficult readability.

**Conclusions:**

Overall, web-based information about induced abortions has difficult readability. Incentives are needed to improve the readability of these texts and ensure that clients encounter understandable information so that they may reach informed decisions and feel adequately prepared when requesting an abortion.

## Background

Worldwide, it is estimated that 56 million induced abortions occur annually, representing approximately 35 abortions per 1000 women [1]. Literature reviews and clinical guidelines emphasize the importance of pre-abortion counseling to offer preparatory information and support clients so that they may reach informed decisions regarding whether or not they want to undergo the abortion and which of the available abortion methods that they prefer [2–4]. Indeed, promoting informed decisions through sufficient and understandable information is essential in reproductive health services [5], including care before an abortion [3]. Those who request an abortion emphasize the importance of feeling sufficiently prepared by receiving sufficient information beforehand. Studies show that clients experience significant fears [6, 7] and uncertainties [6, 8] before an abortion. In order to decrease these fears and promote informed decisions, sufficient preparatory information about abortion is essential [2–4, 6]. It is known that the public now frequently turn towards the Web to access information about induced abortions [7, 9, 10]. If utilized appropriately, the Web has the potential to be a source for accessible information of high quality that may empower the public to reach informed decisions concerning their health [11, 12]. However, the size and structure of the Web implies a risk of contact with information of low quality [11], which may hinder informed decisions and result in ill-prepared patients.

Use of web-based services containing health-related information continues to increase and is today widespread, particularly among younger individuals of reproductive age [13]. The Web is available through handheld devices via mobile networks, with a considerable growth of mobile broadband subscriptions even in the least developed countries [14]. The fact that a high proportion of the public readily have access to the Web has shifted the focus from inequities in physical access to the Internet towards the skills needed to identify and interpret information found on the Web [15]. If web-based information is to reach its full potential, the public need to be able to understand the information that is written, meaning that they need to have sufficient literacy skills [16]. Given the fact that dissemination of health-related information is widespread today, health literacy is an important concept that can have a substantial effect on the lives of those seeking care [17]. Health literacy, defined as the capacity to obtain, process, and understand health-related information well enough to reach decisions [18], has repeatedly been acknowledged as a significant contributor to worsened clinical outcomes and poor use of health care services [19]. However, it is estimated that more than half of the population have intermediate or lower health literacy, and research indicates that as low as 12% have proficient literacy levels [20]. Low health literacy is associated with more hospitalizations, higher mortality and lesser ability to interpret health-related messages [19]. The ability to identify, understand and appraise health-related information from electronic sources is generally poor, even among younger college-level audiences [21].

Considering the aforementioned impacts that low health literacy may have, readability is an important aspect when evaluating the quality of written information developed for the public. Readability is a multidimensional concept that comprises aspects such as typography, the reader’s interest, and the style of writing in the text. The concept is broadly defined by Dale and Chall as ‘the sum total (including the interactions) of all those elements within a given piece of printed material that affects the success that a group of readers have with it’ [22]. In light of the raising use of the Web as a source for health-related information, an increasing number of studies have investigated the readability of web-based information developed for the public. Mainly, these studies focus on assessing the vocabulary and sentence structure with various calculations that illustrate readability levels. Many of these studies report difficult readability levels, which may hinder information uptake among those seeking information about a health-related topic. While studies indicate that web-based information about induced abortions have low quality in regard to various aspects, including information about treatment options, accuracy and comprehensiveness [23–27], little is still known about the readability of such sources.

## Methods

### Aim

The aim of this study was to investigate the readability of web-based information about induced abortion.

### Design

This was a cross-sectional study investigating the readability of web-based information written in the English language, using quantitative readability measures assessing the text-based content. This manuscript follows the STROBE checklist (Additional file 1).

### Identification of web pages

Google, which is the most used online search engine [28], was used to identify web pages about induced abortion. Preliminary search terms were designed by the researchers, based on their understanding of patient’s wordings related to induced abortion. Google Trends was then used to further explore worldwide use of search terms related to abortion, which confirmed the chosen terms. The final search terms used included “*Abortion*”, “*Induced abortion*”, “*Termination of pregnancy*”, “*Terminate a pregnancy*”, “*Abortion pill*”, “*RU-486*”, “*Abortion facts*”, and “*Medical abortion*”. According to previous research, members of the public search for health-related information by screening the first hits in search engines [29–32]. Thus, the first 30 hits were screened for inclusion, resulting in 240 screened hits in total. The searches were performed in September and October 2018. Two links did not work and two links led to web pages written in other languages than English, resulting in 236 hits leading to web pages about induced abortion. Of these, 51 hits led to duplicate web pages, resulting in 185 unique web pages that were included in the sample.

### Data collection

Each included web page was accessed and the text-based content was copied to the web-based readability tool Readable.com, a tool recommended by U.S. National Library of Medicine [33]. The complete set of text from each included web page was imported in the tool and the imported texts were all checked for consistency in comparison with the original text found in each web page. Automated calculations were performed in the tool in regard to the following widely established readability measures: Flesch Kincaid Grade Level (FKGL), Gunning Fog Index (GFI), Coleman-Liau Index (CLI), Simple Measure of Gobbledygook (SMOG), and Flesch Reading Ease (FRE). Four of the measurements (FKGL, GFI, CLI and SMOG) generate a score corresponding to grade levels, which were interpreted as elementary school (1–5), junior high school (6–9) and senior high school or above (≥10). FRE generate a score ranging from 0 to 100, interpreted as easy (80–100), average (60–79) or difficult (0–59). The measurements base the score on different aspects of the number of words, sentences, syllables and letters.

### Data analysis

The data were analyzed with RStudio version 1.0.143 (RStudio, Inc.). Descriptive statistics were calculated to describe the sample, and associations between the readability variables and search rank (i.e. rank in the hit list in the search engine) were calculated with Pearson’s Correlation Coefficient. For comparisons of readability scores between the different affiliations of web pages, the Kruskal-Wallis test was used. Dunn’s test with the bonferroni correction was used for post hoc analysis. In these comparisons, affiliations with < 20 included web pages were excluded due to small sample sizes. *P* < .05 was considered statistically significant.

## Results

The most common affiliations among the included web pages were charities or private organizations (*n* = 50, 27%), while the least common were online shops (*n* = 1, 1%) and churches (n = 1, 1%) (Table 1). Across all grade level measures, a small minority of the included web pages had a readability corresponding to elementary school (*n* ≤ 2, 1%) and the majority had readability corresponding to senior high school or above (*n* ≥ 125, 68%). In total, 157 web pages (85%) had difficult readability according to FRE, i.e. a score between 0 and 59 (Table 2).

**Table 1:** Affiliations among the included web pages (*n* = 185)

Affiliation	n (%)
Charity or private/ patient organization	50 (27.0)
Health care system	35 (18.9)
News/magazine	28 (15.1)
Independent information website	20 (10.8)
Scientific	20 (10.8)
Government	14 (7.6)
Wiki	6 (3.2)
Dictionary	5 (2.7)
University/College	3 (1.6)
Pharmaceutical company	2 (1.1)
Online shop	1 (0.5)
Church	1 (0.5)

**Table 2:** Difficulty levels of the included web pages (n = 185)

Measure	Difficulty level	n (%)
Flesch Kincaid Grade Level	Elementary school (1–5)	2 (1.1)
	Junior high school (6–9)	58 (31.3)
	Senior high school and above (> 10)	125 (67.6)
Gunning Fog Index	Elementary school (1–5)	1 (0.5)
	Junior high school (6–9)	11 (6.0)
	Senior high school and above (> 10)	173 (93.5)
Coleman-Liau Index	Elementary school (1–5)	0 (0.0)
	Junior high school (6–9)	27 (14.6)
	Senior high school and above (> 10)	158 (85.4)
Simple Measure of Gobbledygook	Elementary school (1–5)	0 (0.0)
	Junior high school (6–9)	2 (1.1)
	Senior high school and above (> 10)	183 (98.9)
Flesch Reading Ease	Easy (80–100)	0 (0.0)
	Average (60–79)	28 (15.1)
	Difficult (0–59)	157 (84.9)

**Fig. 1:**
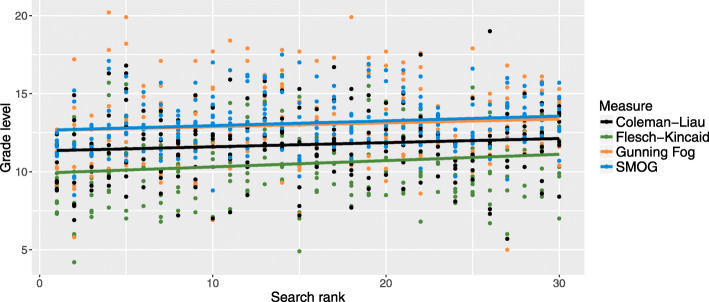
Associations between readability measures and search rank

For the complete sample, the means of the grade level measures ranged between 10.7 and 13.2 (SD 1.8–2.7), indicating that the included web pages had difficult readability levels. Irrespective of grade level measure, the affiliation with the highest mean was scientific sources, and the affiliation with the lowest mean was health care. The mean FRE score was 44.4 (SD 13.5), with lowest mean for scientific sources and highest mean for sources developed and managed by health care. According to the Kruskal-Wallis test, there were significant differences in readability levels between web page affiliations across all reading measures. When comparing web page affiliations with the post hoc test, web pages affiliated with the health care consistently had scores indicating significantly less difficult readability, while web pages affiliated with scientific sources consistently had scores indicating more difficult readability (Table 3). Only weak correlations (r < 0.2) were found between the readability measures and search rank, indicating that readability level is not associated with the list generated by the search engine (Table 4). Although the correlations were weak, all measures increased in grade level as the search rank increased (Fig. 1).

**Table 3:** Readability of the included web pages (n = 185)

Readability measure	Affiliation	Mean (SD)	Range
Flesch Kincaid Grade Level	Charity/private organization^1,2^	10.5 (2.3)	5–15
	Health care^2^	9.1 (2.0)	4–14
	News/magazine^1^	11.2 (2.4)	8–16
	Independent information website^2^	10.6 (2.2)	7–16
	Scientific^1^	12.7 (1.8)	9–16
	Other	11.1 (2.0)	7–15
	All affiliations (complete sample)	10.7 (2.4)	4–16
Gunning Fog Index	Charity/private organization	13.0 (2.7)	5–18
	Health care^2^	11.9 (2.2)	6–16
	News/magazine	13.3 (2.3)	10–18
	Independent information website^2^	13.2 (2.9)	9–20
	Scientific^1^	15.0 (2.4)	10–18
	Other	13.1 (2.7)	9–20
	All affiliations (complete sample)	13.1 (2.7)	5–20
Coleman-Liau Index	Charity/private organization^1,2^	11.6 (2.0)	6–14
	Health care^2^	10.6 (1.9)	7–16
	News/magazine^2^	11.6 (2.0)	7–15
	Independent information website^2^	12.1 (2.3)	8–16
	Scientific^1^	14.4 (1.9)	11–18
	Other	12.5 (2.1)	8–19
	All affiliations (complete sample)	11.9 (2.3)	6–19
Simple Measure of Gobbledygook	Charity/private organization^1^	13.3 (1.8)	9–17
	Health care^2^	12.2 (1.5)	9–16
	News/magazine^1^	13.7 (1.8)	11–18
	Independent information website	12.9 (1.6)	11–17
	Scientific^1^	14.3 (1.8)	11–17
	Other	13.2 (1.8)	10–17
	All affiliations (complete sample)	13.2 (1.8)	9–18
Flesch Reading Ease	Charity/private organization^1,2^	46.3 (10.8)	30–78
	Health care^2^	53.5 (12.3)	22–77
	News/magazine^2^	47.0 (11.8)	23–69
	Independent information website^1,2^	42.0 (15.5)	12–68
	Scientific^1^	30.9 (8.7)	15–51
	Other	39.0 (12.7)	10–64
	All affiliations (complete sample)	44.4 (13.5)	10–78

^1^*P* < .05 compared with web pages affiliated with health care (Dunn’s test); ^2^*P* < .05 compared with web pages affiliated with scientific sources (Dunn’s test)

**Table 4:** Correlation between search rank and readability measures

Readability measure	r	***P***-value
Flesch Kincaid Grade Level	0.15	0.02
Gunning Fog Index	0.08	0.20
Coleman-Liau Index	0.11	0.10
Simple Measure of Gobbledygook	0.15	0.02
Flesch Reading Ease	−0.09	0.14

## Discussion

### Principal results

The aim of this study was to investigate the readability of web-based information about induced abortion. Five widely established measures for determination of readability were used, which consistently showed that the readability was difficult, corresponding to a grade level of senior high school or above. Readability was most difficult for web pages affiliated with scientific sources, while it was least difficult for web pages affiliated with health care. Weak correlation was observed between search rank and readability, indicating that the readability was difficult regardless of search rank in the hit lists.

The findings indicate that text-based information on the Web does not accommodate the needs of persons with literacy levels corresponding to elementary school or junior high school. The literature recommends readability levels below the sixth grade level for patient education materials [34]. The fact that the mean readability levels were higher than 10 across all investigated measures call attention to an imbalance between the literacy of the intended audience for abortion-related information and the readability of the material available they find on the Web. Studies in other health-related fields report similar findings, illustrating that difficult readability levels are widely dispersed across the Web [35–37]. Problematic readability levels are also present in other websites within the field of obstetrics and gynecology [38, 39], further strengthening the results. We argue that there is a considerable need to improve the overall readability levels of text-based information about induced abortions. Website developers need to carefully consider readability when producing content for the public. Improved readability may be achieved through various methods, such as using frequent words, short words [40], illustrations, and a narrative style [41]. This study investigated readability calculated with equations based on words and sentence structure. While it is possible that the included web pages had better readability in regard to other aspects within the concept of readability, including typology and use of illustrations, the results nevertheless illustrate quality deficits in regard to the text-based content.

Clinically, health professionals who provide abortion services need to promote enhanced information uptake among clients seeking an induced abortion. Low health literacy levels are prevalent in the general population [20], meaning that many clients may experience difficulties reading written information. This could result in a lack of psychological preparedness and an increased risk of misunderstandings when seeking an induced abortion, which has been highlighted in previous research exploring the perspectives among women with experience of an abortion [6, 7, 42]. Health professionals who consult clients seeking an induced abortion need to acknowledge the risk of encountering materials of difficult readability when searching for web-based information, perhaps even providing clients readable written information and offering suggestions how to identify high-quality information corresponding to their health literacy levels. Our results emphasize the importance of developing clinical guidelines how to appropriately discuss and inform about web-based information when working in abortion services. Health professionals, researchers and stakeholders should consider initiating multidisciplinary collaborations that aim to improve the readability of high-quality web-based information about induced abortions. When comparing web page affiliation, scientific sources showed the most difficult readability levels and web pages affiliated with health care showed the least difficult readability levels. While this indicate what type of resources clients could benefit the most from reading, these results need to be interpreted with caution. Health professionals who want to refer clients towards certain web-based sources should always aim to assess readability as well as other quality aspects before deciding to recommend a website for clients.

This study contributes with new knowledge that complements previous studies investigating quality of web-based information about induced abortions. Quality of web-based information is multidimensional and concerns many aspects besides readability [43, 44], many of which have been investigated in previous studies. According to previous assessments performed by health professionals and researchers, websites about abortion have considerable quality deficits in regard to comprehensiveness [23, 27], accuracy [25, 45], and transparency [27]. Laypersons also report issues with web-based information about abortions related to quality of information about treatment options, reliability, language, understandability, tone, design, layout, and logic [26]. When our findings are taken together with the results of previous studies, it can be concluded that web-based information about induced abortion has serious quality deficits in regard to many different aspects. Clients who request an abortion are at risk of encountering information that is difficult to comprehend. This can lead to misunderstandings and poor information uptake, calling attention to the need to guide those who plan to use the Web for supplemental information towards understandable sources of high quality.

### Methodological considerations

We performed searches designed to mimic the search patterns of the public, using the most used search engine online and exploring Google Trends to validate our search terms. Reports indicate that over 80% of those who search for information via the Web use Google, a number which continues to rise in recent years and reaching as high as 97% of the population in some areas [46, 47]. In light of these statistics, we argue that only using Google could have produced more generalizable results in line with what most Internet users encounter. Nevertheless, it is possible that a proportion of the population decide to use other search engines and the results need to be interpreted with this in mind. We included the first 30 hits retrieved in the list from the search engine, which by far represent the limited number of hits usually accessed by the public when searching for health-related information [29–32].

Readability was determined with an online tool recommended by U.S. National Library of Medicine [33] and which has been used in previous reports [48, 49]. The readability was determined through a series of widely established automated calculations. These measures are efficient in determining readability in regard to quantitative variables based on calculations of texts, involving the number of and relationship between words, sentences, letters, and syllables. However, the tests do not take into consideration complex aspects related to the readability, such as use of words that few may recognize including medical terminology. Readability formulas have been criticized as problematic due to the potentially simplistic approach, as they are based on counting formal properties in texts [50]. For a comprehensive understanding of readability that complements the results of this study, more studies that explores how the intended audience experience abortion-related information is needed.

## Conclusion

The readability of web-based information about induced abortions is difficult, corresponding to senior high school or above. The difficult readability is found irrespective of search rank in the hit list retrieved in the search engine. Members of the public who search for supplemental information about abortions are at risk of encountering information that is difficult to understand, possibly leading to misunderstandings, impaired decision-making, and insufficient preparatory information. Incentives that aim to improve the readability of web-based sources about abortions are needed. Health professionals who consult those who request an abortion should address the identified quality deficits and guide clients towards high-quality sources that contain readable and understandable information.

## Supplementary information

**Additional file 1.** STROBE checklist for cross-sectional studies.

## Data Availability

The datasets used and/or analyzed during the current study are available from the corresponding author on reasonable request.

## Abbreviations

FKGL: Flesch Kincaid Grade Level
GFI: Gunning Fog Index
CLI: Coleman-Liau Index
SMOG: Simple Measure of Gobbledygook
FRE: Flesch Reading Ease
